# Supplementary material for: The Systems Biology Research Tool: evolvable open-source software
Source: BMC Syst Biol. 2008 Jun 29;2:55. doi: 10.1186/1752-0509-2-55 (PMC2446383; doi:10.1186/1752-0509-2-55)
Supplement: Additional file 1 — SBRT Archive. An archive of the current version of the Systems Biology Research Tool. [file 1752-0509-2-55-S1.zip › sbrt-1.4.0/doc/developers_guide/api/sbrt/kernel/fba/comp/AbstractCvComputer.html]

AbstractCvComputer


|  |  |  |
| --- | --- | --- |
| **Overview** \| **Package** \| **Class** \| **Use** \| **Tree** \| **Deprecated** \| **Index** \| **Help** | |  |
| PREV CLASS   **NEXT CLASS** | **FRAMES**    **NO FRAMES**     **All Classes** |  |
| SUMMARY: NESTED \| FIELD \| CONSTR \| METHOD | DETAIL: FIELD \| CONSTR \| METHOD |  |


---


## sbrt.kernel.fba.comp Class AbstractCvComputer

```
java.lang.Object
  sbrt.kernel.fba.comp.AbstractCvComputer
```

**All Implemented Interfaces:**: FluxCvComputer

**Direct Known Subclasses:**: AbstractFluxIntervalComputer, CdHarFluxVectorComputer, RxnDeletionComputer

---

``` public abstract class AbstractCvComputer extends java.lang.Object implements FluxCvComputer ```

This abstract class is a skeleton implementation of `FluxCvComputer`.

**Author:**
:   This class was written and documented by Jeremiah Wright
    while in the Wagner lab.

---

**Constructor Summary**

`AbstractCvComputer(IrrevRxnFluxome fluxome)`
  
          Constructs a new constraint-variation computer.


| **Method Summary** | |
| --- | --- |
| `protected  void` | `checkFluxConstraint(Interval c, java.lang.String rxnName, double tolerance)`             Ensures the flux constraint is valid for the fluxome provided. |
| `protected  void` | `checkNumber(double number)`             Ensures the provided number is not `NaN` or infinite. |
| `protected  void` | `checkRxnExpr(MathExpr<java.lang.String> e)`             Ensures the expression is valid for the fluxome provided. |
| `protected  void` | `checkRxnExprConstraint(Interval c)`             Ensures the expression constraint is valid for the fluxome provided. |
| `protected  void` | `checkRxnName(java.lang.String reactionName)`             Ensures the reaction name is valid for the fluxome provided. |
| `protected  void` | `checkRxnNames(java.util.Collection<java.lang.String> reactionNames)`             Ensures the reaction names are valid for the fluxome provided. |
| `IrrevRxnFluxome` | `getFluxome()`             Returns the fluxome for which values will be computed. |
| `protected  LinearComb<java.lang.String>` | `getRxnExpr(java.util.Collection<java.lang.String> reactionNames)`             Converts a collection of reaction names to a linear combination. |
| `protected  LinearComb<java.lang.String>` | `getRxnExpr(java.lang.String reactionName)`             Converts a reaction name to a single-term linear combination. |
| `void` | `restoreFluxConstraints()`             Restores the flux constraints for all reactions in the fluxome to those values contained in the fluxome. |
| `void` | `restoreFluxConstraints(java.util.Collection<java.lang.String> reactionNames)`             Restores the flux constraints for the provided reaction names to the values contained in the fluxome. |
| `void` | `restoreRxnExprConstraints(java.util.Collection<? extends MathExpr<java.lang.String>> rxnNameExps)`             Restores the constraints on the provided expressions to the values contained in the fluxome. |
| `void` | `setFluxConstraints(java.util.Map<java.lang.String,Interval> fluxConstraints)`             Changes the flux constraints on more than one reaction. |
| `void` | `setRxnExprConstraints(java.util.Map<? extends MathExpr<java.lang.String>,Interval> constraints)`             Changes the constraints on more than one mathematical expression of fluxes. |

**Methods inherited from class java.lang.Object**

`clone, equals, finalize, getClass, hashCode, notify, notifyAll, toString, wait, wait, wait`

**Methods inherited from interface sbrt.kernel.fba.comp.FluxCvComputer**

`getFluxConstraint, getFluxConstraints, getRxnExprConstraint, getRxnExprConstraints, restoreFluxConstraint, restoreRxnExprConstraint, restoreRxnExprConstraints, setFluxConstraint, setRxnExprConstraint`

**Constructor Detail**

### AbstractCvComputer

```
public AbstractCvComputer(IrrevRxnFluxome fluxome)
```

:   Constructs a new constraint-variation computer.

    **Parameters:**: `fluxome` - the fluxome for which values will be computed. **Throws:**: `java.lang.NullPointerException` - if the provided fluxome is null.


**Method Detail**

### getFluxome

```
public IrrevRxnFluxome getFluxome()
```

:   Returns the fluxome for which values will be computed.

    :   **Specified by:**: `getFluxome` in interface `FluxCvComputer`
    :   **Returns:**: the fluxome for which values will be computed.

---


### checkRxnName

```
protected void checkRxnName(java.lang.String reactionName)
```

:   Ensures the reaction name is valid for the fluxome provided.

    :   **Parameters:**: `reactionName` - String The reaction name to check. **Throws:**: `java.lang.IllegalArgumentException` - if the reaction name is not present in the fluxome.

---


### checkRxnNames

```
protected void checkRxnNames(java.util.Collection<java.lang.String> reactionNames)
```

:   Ensures the reaction names are valid for the fluxome provided.

    :   **Parameters:**: `reactionNames` - the reaction names to check. **Throws:**: `java.lang.IllegalArgumentException` - if a reaction name in the provided collection is not present in the fluxome.: `java.lang.NullPointerException` - if the provided Collection is `null`.

---


### checkRxnExpr

```
protected void checkRxnExpr(MathExpr<java.lang.String> e)
```

:   Ensures the expression is valid for the fluxome provided.

    :   **Parameters:**: `e` - the expression to check. **Throws:**: `java.lang.IllegalArgumentException` - if the expression contains variables that are not valid reaction names in the fluoxme.: `java.lang.NullPointerException` - if the provided expression is `null`.

---


### checkFluxConstraint

```
protected void checkFluxConstraint(Interval c,
                                   java.lang.String rxnName,
                                   double tolerance)
```

:   Ensures the flux constraint is valid for the fluxome provided.

    :   **Parameters:**: `c` - the constraint to check.: `rxnName` - the name of the reaction whose flux this constraint applies to.: `tolerance` - the numerical tolerance to use during the comparison. **Throws:**: `java.lang.IllegalArgumentException` - if the flux constraint is not valid for the fluoxme provided.: `java.lang.NullPointerException` - if the provided constraint is `null`.

---


### checkRxnExprConstraint

```
protected void checkRxnExprConstraint(Interval c)
```

:   Ensures the expression constraint is valid for the fluxome provided.

    :   **Parameters:**: `c` - the constraint to check. **Throws:**: `java.lang.NullPointerException` - if the provided constraint is `null`.

---


### checkNumber

```
protected void checkNumber(double number)
```

:   Ensures the provided number is not `NaN` or infinite.

    :   **Parameters:**: `number` - the number to check. **Throws:**: `java.lang.IllegalArgumentException` - if the provided number is `NaN` or infinite.

---


### getRxnExpr

```
protected LinearComb<java.lang.String> getRxnExpr(java.lang.String reactionName)
```

:   Converts a reaction name to a single-term linear combination.

    :   **Parameters:**: `reactionName` - the reaction name to convert. **Returns:**: a linear combination containing a single term, with the provided reaction name as the variable and coefficient equal to 1.

---


### getRxnExpr

```
protected LinearComb<java.lang.String> getRxnExpr(java.util.Collection<java.lang.String> reactionNames)
```

:   Converts a collection of reaction names to a linear combination.

    :   **Parameters:**: `reactionNames` - the reaction names to appear as variables. **Returns:**: a linear combination of the provided reaction names.

---


### setRxnExprConstraints

```
public void setRxnExprConstraints(java.util.Map<? extends MathExpr<java.lang.String>,Interval> constraints)
```

:   Changes the constraints on more than one mathematical expression
    of fluxes.

    :   **Specified by:**: `setRxnExprConstraints` in interface `FluxCvComputer`
    :   **Parameters:**: `constraints` - mathematical expressions of reaction names mapped to their respective constraints.

---


### setFluxConstraints

```
public void setFluxConstraints(java.util.Map<java.lang.String,Interval> fluxConstraints)
```

:   Changes the flux constraints on more than one reaction.

    :   **Specified by:**: `setFluxConstraints` in interface `FluxCvComputer`
    :   **Parameters:**: `fluxConstraints` - reaction names mapped to their respective flux constraints.

---


### restoreRxnExprConstraints

```
public void restoreRxnExprConstraints(java.util.Collection<? extends MathExpr<java.lang.String>> rxnNameExps)
```

:   Restores the constraints on the provided expressions to the values
    contained in the fluxome.

    :   **Specified by:**: `restoreRxnExprConstraints` in interface `FluxCvComputer`
    :   **Parameters:**: `rxnNameExps` - the reaction name expressions whose contraints are to be restored.

---


### restoreFluxConstraints

```
public void restoreFluxConstraints(java.util.Collection<java.lang.String> reactionNames)
```

:   Restores the flux constraints for the provided reaction names to the
    values contained in the fluxome.

    :   **Specified by:**: `restoreFluxConstraints` in interface `FluxCvComputer`
    :   **Parameters:**: `reactionNames` - the reaction names whose flux constraints are to be restored.

---


### restoreFluxConstraints

```
public void restoreFluxConstraints()
```

:   Restores the flux constraints for all reactions in the fluxome to those
    values contained in the fluxome.

    :   **Specified by:**: `restoreFluxConstraints` in interface `FluxCvComputer`


---


|  |  |  |
| --- | --- | --- |
| **Overview** \| **Package** \| **Class** \| **Use** \| **Tree** \| **Deprecated** \| **Index** \| **Help** | |  |
| PREV CLASS   **NEXT CLASS** | **FRAMES**    **NO FRAMES**     **All Classes** |  |
| SUMMARY: NESTED \| FIELD \| CONSTR \| METHOD | DETAIL: FIELD \| CONSTR \| METHOD |  |


---
